# Supplementary material for: 3,4-Dichlorophenylacetic acid acts as an auxin analog and induces beneficial effects in various crops
Source: Commun Biol. 2024 Feb 8;7:161. doi: 10.1038/s42003-024-05848-9 (PMC10853179; doi:10.1038/s42003-024-05848-9)
Supplement: Supplementary file 2 — SUPPLEMENTAL MATERIAL [file 42003_2024_5848_MOESM2_ESM.pdf]

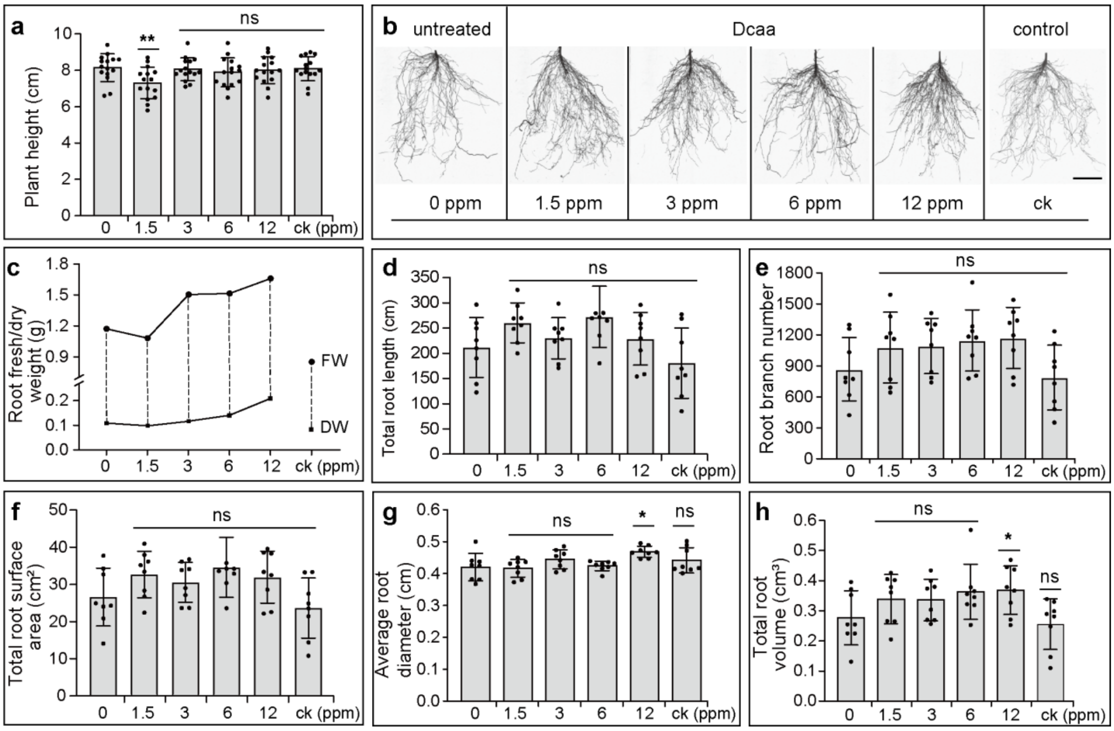

Supplementary Figure 1. Dcaa promotes the growth of cabbage roots.

a and **b** The plant height (**a**) and root systems (**b**) of cabbage plants 7 days after being treated with various concentrations of Dcaa and a plant growth regulator composed of 1 ppm potassium indole butyrate and 1 ppm sodium naphthalene acetate (ck). **c-h** Root fresh/dry weight (**c**), total root length (**d**), root branch number (**e**), total root surface area (**f**), average root diameter (**g**), and total root volume (**h**). Error bars represent the SD of the mean of 8 to 15 cabbage plants. * $P < 0.05$, ** $P < 0.01$, *ns* not significant (Student's *t*-test, two-tailed, two-sample equal variance). Bar = 2 cm (**b**).

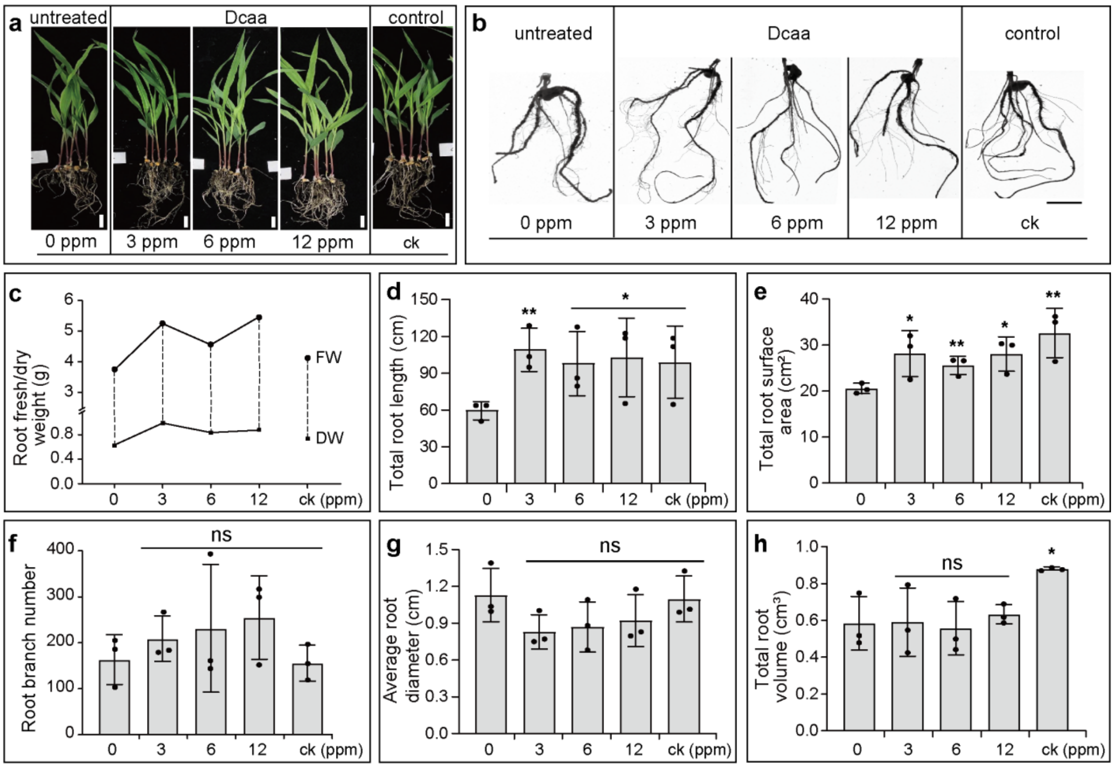

Supplementary Figure 2. Dcaa promotes the growth of maize roots.

a and **b** Representative images of maize seedlings (**a**) and root systems (**b**) 7 days after being treated with various concentrations of Dcaa and a plant growth regulator composed of 1 ppm potassium indole butyrate and 1 ppm sodium naphthalene acetate (ck). **c-h** Root fresh/dry weight (**c**)**,** total root length (**d**), total root surface area (**e**), root branch number (**f**)**,** average root diameter (**g**), and total root volume (**h**). Error bars represent the SD of the mean of 3 maize plants. * $P < 0.05$, ** $P < 0.01$, *ns* not significant (Student's *t*-test, one-tailed, two-sample equal variance). Bars = 2 cm in (**a**) and (**b**).

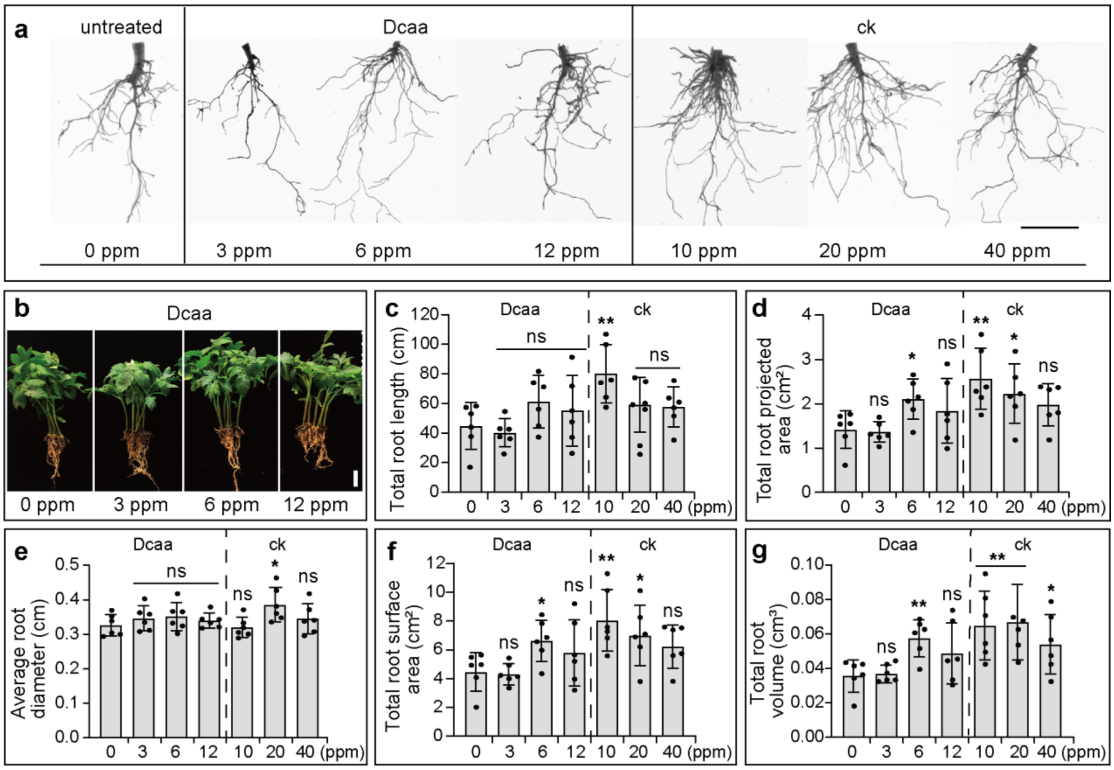

Supplementary Figure 3. Dcaa promotes the growth of tomato roots.

a and **b** Representative images of the root system (**a**) and the whole plant (**b**) of tomato plants 7 days after being treated with various concentrations of Dcaa and a plant growth regulator composed of potassium indole butyrate and sodium naphthalene acetate (ck). **c-g** Total root length (**c**), total root projected area (**d**), average root diameter (**e**), total root surface area (**f**), and total root volume (**g**). Error bars represent the SD of the mean of 6 tomato plants. * $P < 0.05$, ** $P < 0.01$, *ns* not significant (Student's *t*-test, two-tailed, two-sample equal variance). Bars = 2 cm in (**a**) and (**b**).

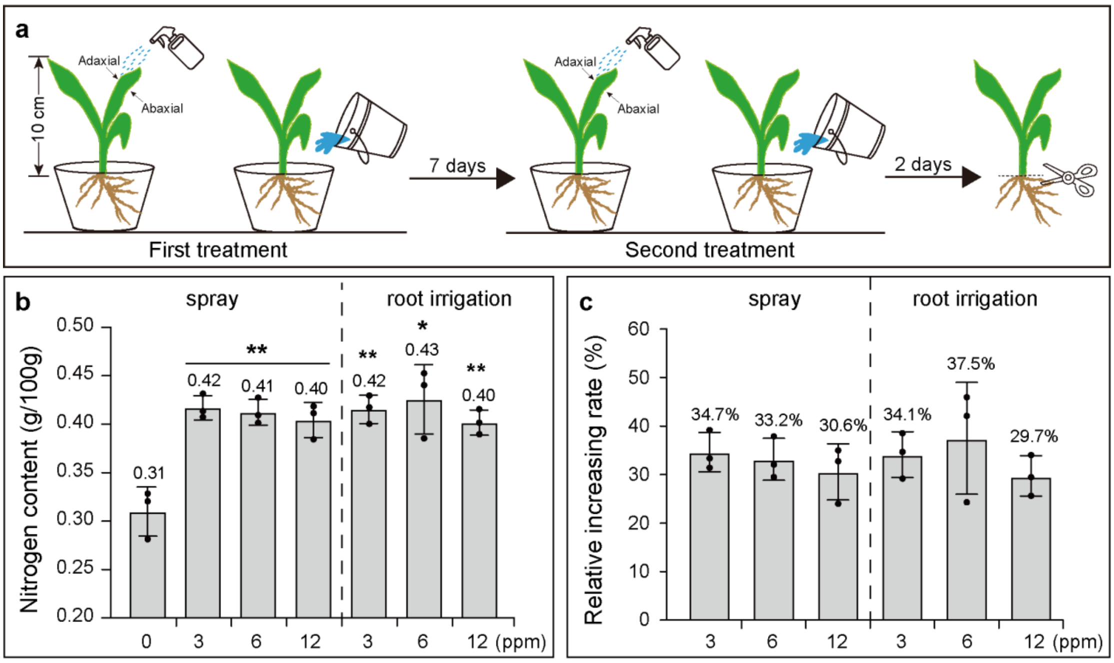

Supplementary Figure 4. Dcaa promotes nitrogen fertilizer use efficiency in maize.

a Schematic diagram of determination of nitrogen use efficiency in maize. **b** and **c** Nitrogen content (b) and relative increasing rate (**c**) in leaves of maize seedlings after spraying or irrigating with various concentrations of Dcaa. Error bars represent the SD of the mean of three biological experiments. * P < 0.05, ** P < 0.01 (Student's *t*-test, two-tailed, two-sample equal variance).

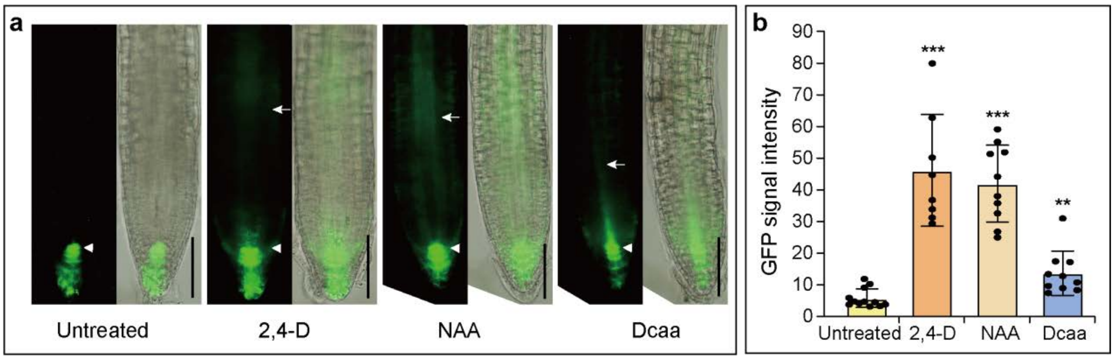

Supplementary Figure 5. Dcaa enhances expression of the *DR5rev:GFP* auxin-responsive reporter.

a *DR5rev:GFP* fluorescence signals in the root tip of 7-day-old Arabidopsis seedlings untreated or treated with 10 μM 2,4-D and NAA and 500 μM Dcaa. Triangles indicate the QC (quiescent center); white arrows indicate the GFP signals at the root elongation zone (in 2,4-D and NAA treated roots) and transition zone (in the Dcaa treated root). Bars = 100 μm. **b** Quantitative *DR5rev:GFP* fluorescence intensity in the root tip of 7-day-old seedlings. Error bars represent the SD of the mean of 8-12 seedlings. ** $P < 0.01$, *** $P < 0.001$ (Student's *t*-test, two-tailed, two-sample equal variance).

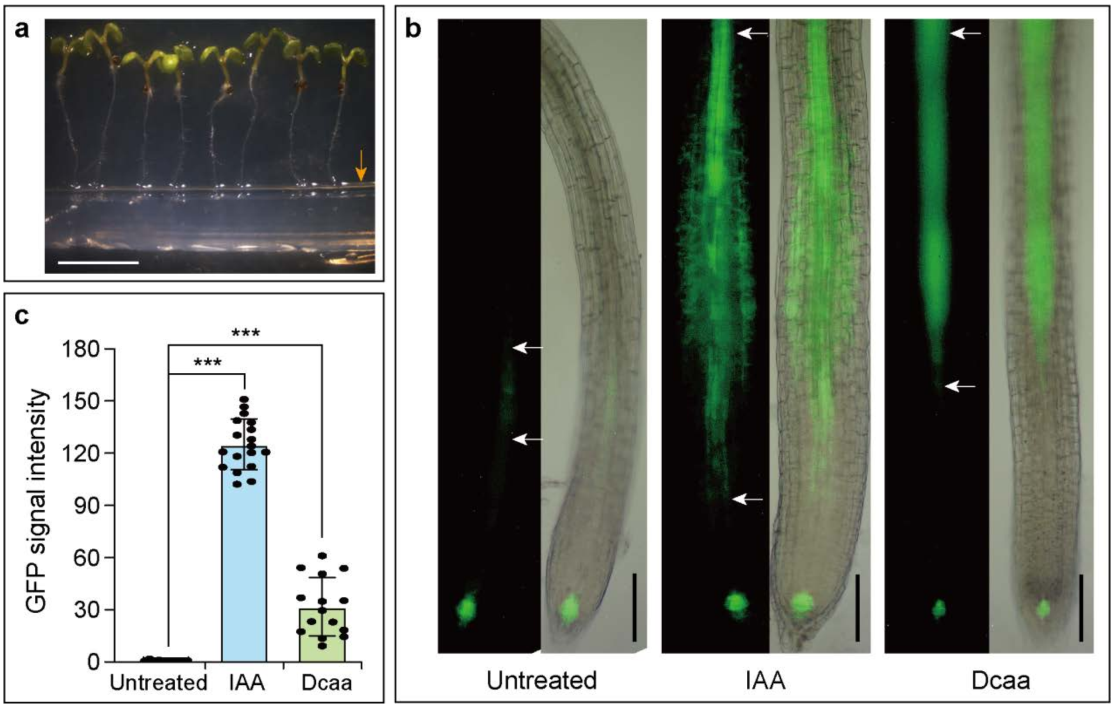

Supplementary Figure 6. Application of IAA and Dcaa at the root tip of the *DR5rev:GFP* seedlings induces *GFP* expression at the root elongation zone and differentiation zone.

a The image shows the Dcaa containing agar gel strip placed below the root tip (overlap with the root tip by approximately 0.2 mm) of 5-day-old vertically grown *DR5rev:GFP* seedlings. The orange arrow indicates the gel strip. **b** and **c** *DR5rev:GFP* fluorescence intensity along the root tip towards the differentiation zone of the primary root 13 h after agar strips containing 0 (untreated), 100 μM IAA, or 1.5 mM Dcaa were placed below the root tip. White arrows indicate the GFP signals at the root elongation zone or transition zone. Error bars in **c** represent the SD of the mean of 10-18 roots. *** $P < 0.001$ (Student's *t*-test, two-tailed, two-sample equal variance). Bars = 1 cm (**a**) and 100 μm (**b**).

Supplementary Table 1. List of primers used in this research.

Primer name	Primer sequence 5'–3'
IAA19F	ATGGAGAAGGAAGGACTCGGGCTTG
IAA19R	GTCTTCGTATATGGTAACGTATTCGC
ARF7F	TCAAGGTCACAGTGAGCAAGTCG
ARF7R	TGTGGAGCATGCATATGAGCTTGG
ARF19F	TGAAACTAAAGGCCCTGCACAAGC
ARF19R	TCCAACGAAGGAGAGAAGAAGCCA
LBD16F	TGCCCCTGTTTATGGATGTGTC
LBD16R	TGATTGCAAGAAAGCCACCT
SAUR22F	TTTTGTGCAACCGACACGAC
SAUR22R	TCGACGTGCTACCATTGAGG
SAUR24F	GAGATATTTGGTGCCTGTCTCATATTTAAACC
SAUR24R	CAAGAAGAAAGAGGAAAAAGGGCTCATC
TIP41F	GTATGAAGATGAACTGGCTGACAAT
TIP41R	ATCAACTCTCAGCCAAAATCGCAAG
