# Supplementary material for: 3,4-Dichlorophenylacetic acid acts as an auxin analog and induces beneficial effects in various crops
Source: Commun Biol. 2024 Feb 8;7:161. doi: 10.1038/s42003-024-05848-9 (PMC10853179; doi:10.1038/s42003-024-05848-9)
Supplement: Supplementary file 3 — Description of Additional Supplementary Files [file 42003_2024_5848_MOESM3_ESM.pdf]

Description of Additional Supplementary Files

File name: Supplementary Data 1

Description: The source data behind the graphs in the paper and the Bioactivity assay of auxin analogues.
